# Right-left ventricular shape variations in tetralogy of Fallot: associations with pulmonary regurgitation

**DOI:** 10.1186/s12968-021-00780-x

**Published:** 2021-10-07

**Authors:** Charlène A. Mauger, Sachin Govil, Radomir Chabiniok, Kathleen Gilbert, Sanjeet Hegde, Tarique Hussain, Andrew D. McCulloch, Christopher J. Occleshaw, Jeffrey Omens, James C. Perry, Kuberan Pushparajah, Avan Suinesiaputra, Liang Zhong, Alistair A. Young

**Affiliations:** 1 Department of Anatomy and Medical Imaging, University of Auckland, Auckland, New Zealand; 2 Auckland Bioengineering Institute, University of Auckland, Auckland, New Zealand; 3 University of California San Diego, La Jolla, CA USA; 4 University of Texas Southwestern Medical Centre, Dallas, TX USA; 5 Inria, Palaiseau, France; 6 LMS, École Polytechnique, CNRS, Institut Polytechnique de Paris, Palaiseau, France; 7 Department of Mathematics, Faculty of Nuclear Sciences and Physical Engineering, Czech Technical University in Prague, Prague, Czech Republic; 8 Division of Cardiology, Rady Children’s Hospital, San Diego, CA USA; 9 Department of Cardiology, Auckland District Health Board, Auckland, New Zealand; 10 Department of Biomedical Engineering, King’s College London, London, UK; 11 School of Computing, University of Leeds, Leeds, UK; 12 National Heart Centre, Singapore, Singapore; 13 Duke-NUS Medical School, Singapore, Singapore

**Keywords:** Cardiovascular magnetic resonance, Ventricular function, Atlases, Myocardial deformation, Tetralogy of Fallot

## Abstract

**Background:**

Relationships between right ventricular (RV) and left ventricular (LV) shape and function may be useful in determining optimal timing for pulmonary valve replacement in patients with repaired tetralogy of Fallot (rTOF). However, these are multivariate and difficult to quantify. We aimed to quantify variations in biventricular shape associated with pulmonary regurgitant volume (PRV) in rTOF using a biventricular atlas.

**Methods:**

In this cross-sectional retrospective study, a biventricular shape model was customized to cardiovascular magnetic resonance (CMR) images from 88 rTOF patients (median age 16, inter-quartile range 11.8–24.3 years). Morphometric scores quantifying biventricular shape at end-diastole and end-systole were computed using principal component analysis. Multivariate linear regression was used to quantify biventricular shape associations with PRV, corrected for age, sex, height, and weight. Regional associations were confirmed by univariate correlations with distances and angles computed from the models, as well as global systolic strains computed from changes in arc length from end-diastole to end-systole.

**Results:**

PRV was significantly associated with 5 biventricular morphometric scores, independent of covariates, and accounted for 12.3% of total shape variation (p < 0.05). Increasing PRV was associated with RV dilation and basal bulging, in conjunction with decreased LV septal-lateral dimension (LV flattening) and systolic septal motion towards the RV (all p < 0.05). Increased global RV radial, longitudinal, circumferential and LV radial systolic strains were significantly associated with increased PRV (all p < 0.05).

**Conclusion:**

A biventricular atlas of rTOF patients quantified multivariate relationships between left–right ventricular morphometry and wall motion with pulmonary regurgitation. Regional RV dilation, LV reduction, LV septal-lateral flattening and increased RV strain were all associated with increased pulmonary regurgitant volume. Morphometric scores provide simple metrics linking mechanisms for structural and functional alteration with important clinical indices.

**Supplementary Information:**

The online version contains supplementary material available at 10.1186/s12968-021-00780-x.

## Background

The survival rate of repaired tetralogy of Fallot (rTOF) patients has greatly increased due to the improvement of surgical repair. Currently, the early mortality rate is below 2% [1] and the 25-year survival rate is above 94% [2]. However, pulmonary regurgitation (PR) is a common consequence of the surgical relief of right ventricular (RV) outflow tract narrowing. rTOF patients are therefore monitored for PR, and its effects on RV dysfunction and long-term outcomes [3, 4]. Despite being well tolerated in childhood, PR has damaging effects on RV dysfunction and leads to RV dilation over an ill-defined period of time. In follow-up, rTOF patients may demonstrate progressive exercise intolerance, arrhythmia, right- or left-sided heart failure, RV and/or left ventricular (LV) dysfunction or sudden cardiac death [5–7]. Patients with a history of severe PR are at risk for irreversible RV functional impairment making timing for pulmonary valve replacement a critical issue [8, 9]. However, the timing of valve replacement based on RV size and systolic function remains a subject of debate, with wide inter-institutional, qualitative variations of clinical practice [3, 4, 10, 11].

Studies of RV remodeling in rTOF have demonstrated a strong relationship between RV dilation, RV bulging, and apical dilation with the presence of pulmonary regurgitation [12–14]. However, the relationships with LV function and the underlying mechanisms are less understood in rTOF population and few studies have focused on RV-and-LV interactions [15–19]. Increased RV dilation has been known to affect LV function in rTOF [19–22] and approximately 20% of adult rTOF patients develop LV dysfunction [23]. Furthermore, the close relationship between LV ejection fraction (LVEF) and RV ejection fraction (RVEF) has been previously demonstrated [24, 25], indicating the importance of ventricular interactions in rTOF. This has led to the suggestion that LV function and inter-ventricular interactions should be considered in the timing of pulmonary valve replacement [26, 27]. Conventional clinical metrics of ventricular volume and function remain limited in their scope and do not fully explain the contribution of biventricular shape and interaction, thus limiting the value of imaging data in clinical evaluation and understanding of pathophysiology in relation to clinical outcomes.

Here, we use a biventricular atlas [28] to quantify relationships between RV and LV shape and function, and examine their associations with PR, in a retrospective cross-sectional study of 88 rTOF patients with no history of pulmonary valve replacement. We aimed to determine whether novel 3D shape features, calculated from standard 3D imaging exams, can be used to quantify specific shape and function alterations associated with clinically important metrics such as PR. If so, these methods could be readily applied to any cross-sectional imaging exam to provide shape scores in relation to factors of interest, thereby providing a mechanistic link between these factors and alterations in cardiac geometry and function and reducing inherent inaccuracies of qualitative clinical decision-making.

## Methods

### Study population

Cardiovascular magnetic resonance (CMR) examinations and clinical data from 88 rTOF patients were obtained from the Cardiac Atlas Project (CAP) congenital heart disease database [29].

CAP is a large-scale database of cardiac images and limited associated clinical data that facilitates data sharing for the development of new methods for image analysis and collaborative statistical analysis of heart shape and function across multiple cohorts (cardiacaltas.org). Datasets related to congenital heart disease were added to the CAP beginning in 2015.

In this paper, deidentified datasets were contributed from two clinical centers (Auckland, New Zealand and San Diego, California, United States) with approval from local institutional review boards compatible with data sharing. Demographic data are shown in Table 1. Patients with pulmonary valve replacement, or severe tricuspid regurgitation from either echocardiography or CMR were excluded.

**Table 1:** Characteristics of the 88 rTOF participants

Variables	N = 88
Age at CMR scan (y)	16 (11.8, 24.3)
Sex (F/M)	35/53
Height (cm)	160 (149.8, 168)
Weight (kg)	58.3 ± 25.4
PRF (%)	36.9 ± 14.4
PRV_i_ (ml/m^2^)	23.7 (14.2, 33.3)
Age at primary repair (y)	0.8 (0.25, 1.6)
Time after primary repair (y)	15.7 (10.9, 21)
BMI (kg/m^2^)	23.0 (17.8, 26.5)
Tricuspid regurgitation*:	
None-trace	57 (64.8%)
Mild	18 (20.4%)
Mild-to-moderate	6 (6.8%)
Moderate	7 (8.0%)
Severe	0
Numbers with reintervention	33 (37.5%)
Pulmonary valve replacement	0
Types of repair:	
Transannular patch	69 (78.4%)
Valve-Sparing	5 (5.7%)
Conduit	14 (15.9%)

Normally distributed data are presented as mean ± std. dev and median (interquartile ranges) otherwise. *PRF* pulmonary regurgitant fraction, *PRVI*i pulmonary regurgitant volume index, *BMI* body mass index. *Tricuspid regurgitation from the MRI report

### CMR imaging protocol and image analysis

CMR images were acquired with either prospectively or retrospectively electrocardiogram (ECG) gated balanced steady-state free-precession cine sequence on 1.5 T CMR scanners (Avanto, Siemens Healthineers, Erlangen, Germany, or Discovery MR450, GE Healthcare Systems, Chicago, Illinois, United States) during breath-holding. The short axis slices were acquired parallel to the tricuspid annulus plane and spanned both ventricles. Long-axis slices were obtained through all valves in standard 4-chamber, 2-chamber, LV outflow tract and RV outflow tract views. Typical imaging parameters were: repetition time 24-32 ms; echo time 1.1–1.5 ms; flip angle 70–80°; pixel size reconstructed to in plane 0.59–1.75 $\times$ 0.59–1.75 mm; slice thickness 4–6 mm; number of time frames 20–35; image matrix 180–224 $\times$ 208–256, and field of view 200–300 mm.

Antegrade and retrograde pulmonary flow measurements were obtained from two-dimensional phase contrast (2D PC) imaging. PC analysis of antegrade and retrograde flows in the main artery was performed on a plane at a location just below the pulmonary artery (PA) bifurcation and perpendicular to the axis of the PA. Typical imaging parameters were: acceleration factor 3, echo time = 2.3–3.0 ms, repetition time = 4.8–5.0 ms, field of view = 169–315 mm × 300–420 mm, spatial resolution = (1.4–2.0) × (1.4–2.0) × (5–8) mm^3^, temporal resolution = 37–41 ms, flip angle of 15°-30°. Scouts were used to set the velocity encoding.

Contours were drawn manually on both long axis and short axis cine slices by one expert analyst using Segment [30]. Contours from another independent expert analyst were also performed for 35 cases. Ventricular masses and volumes at end-diastole (ED) and end-systole (ES) for both ventricles were calculated using the volumetric summation of discs method. Papillary muscles were excluded from the masses and endocardial contours segmentation. Tricuspid and mitral valve hinge points were defined from the intersection of the left atrial and ventricular contours delineated on the 2-chamber and 4-chamber long axis images, and right atrial and ventricular contours on the 4-chamber long axis images. Aortic valve hinge points were extracted from the LV outflow images and the ventricular extent of the RV outflow tract was extracted from the RV outflow tract images. When aortic and pulmonary leaflets were not visible, boundary points were defined by the transition in appearance from myocardium to vessel wall.

### Biventricular atlas

**Fig. 1:**
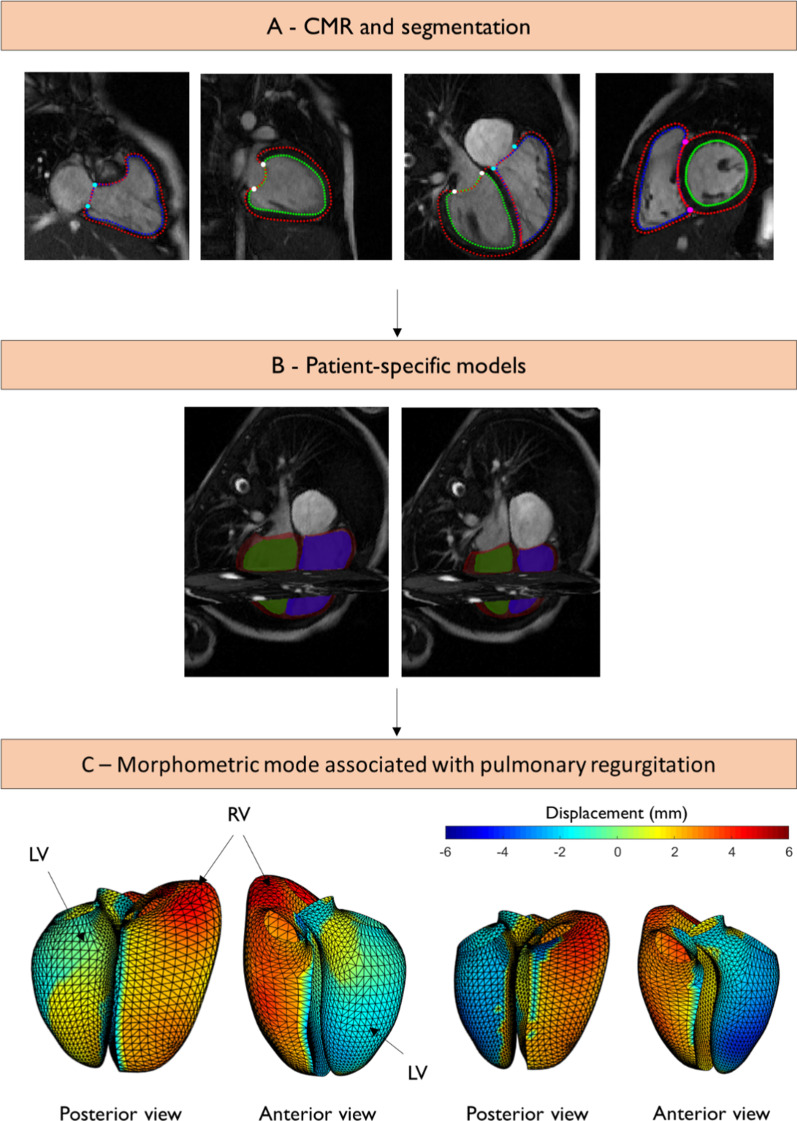
Overview of the biventricular atlas construction. **A** Contours and landmarks on short and long axis cardiovascular magnetic resonance (CMR) images at end-diastole; **B** model fit at end-diastole (ED) (left) and end-systole (ES) (right). The left ventricle (LV) is shown in green, the right ventricle (RV) is shown in blue and the epicardium is shown in maroon; **C** endocardial surface colors showing differences in shape at ED (left) and ES (right) from the mean (25.5 ml/m^2^) to high (40 ml/m^2^) pulmonary regurgitant volume index (PRVI). Scale bar is in mm. Red denotes displacement outward from the LV, and blue denotes displacement inward to the LV

A biventricular subdivision surface template mesh was constructed as described previously [28]. An overview of the analysis pipeline is shown in Fig. 1. This included the LV, RV, and the four valves (aortic, mitral, pulmonary, and tricuspid). The template mesh was automatically customized to each patient and breath-hold slice misregistration was also automatically corrected using an iterative registration algorithm [31]. Valve locations were customized to the manual landmarks by using landmark registration and surfaces were customized by using diffeomorphic non-rigid registration to the manual contours. LV and RV volume and mass were calculated by numerical integration of mesh volumes. To build the biventricular atlas, all the ED mesh points were first aligned to the mean mesh surface points by a rigid registration (translation and rotation). This transformation was then applied to the ES mesh. The ED and ES surface points were then concatenated to form a single combined shape. Principal component analysis (PCA) was used to evaluate the distribution of shape variation across the cohort [32]. This results in a relatively small number of components (24 in this study) that describe the shape variation across all patients, while accounting for correlations between points in the model. The first component explains the most variance, the second component explains the most remaining variance, and so on. For each patient, morphometric scores were calculated, which quantified the amount of each component present (“Appendix”). Owing to the combination of ED and ES shapes in each mode, both shape and shape changes between ED and ES could be captured using this method.

### Pulmonary regurgitant volume

Pulmonary regurgitation was quantified from CMR imaging using pulmonary regurgitant volume indexed to body surface area (PRVI) as this has been shown to be a more accurate reflection of regurgitation severity compared with pulmonary regurgitant fraction [12, 33–35]. PR was quantified using PC imaging, and forward and regurgitant flows in the main PA were quantified using commercially available software (Argus Flow, Siemens Healthineers, and cvi42, Circle Cardiovascular Imaging, Calgary, Alberta, Canada). The range of PRVI was 1.5–95.7 ml/m^2^ with median of 23.7 and IQR (14.2, 33.3) ml/m^2^ (Table 1).

### Multivariate associations with pulmonary regurgitant volume

A linear regression model was constructed to estimate associations with biventricular heart shape, using the morphometric scores as the response (dependent) variables [28]. PRVI, height, weight and age were included as continuous predictor variables, and sex and tricuspid regurgitation severity from the CMR report (none/mild/mild-to-moderate/moderate) were included as categorical predictors. Height, weight, sex, and age were added to the model to control for body habitus as done previously [28]. LV and RV volumes and mass could be calculated from the resulting morphometric scores, as well as LV and RV dimensions.

### Shape features

**Fig. 2:**
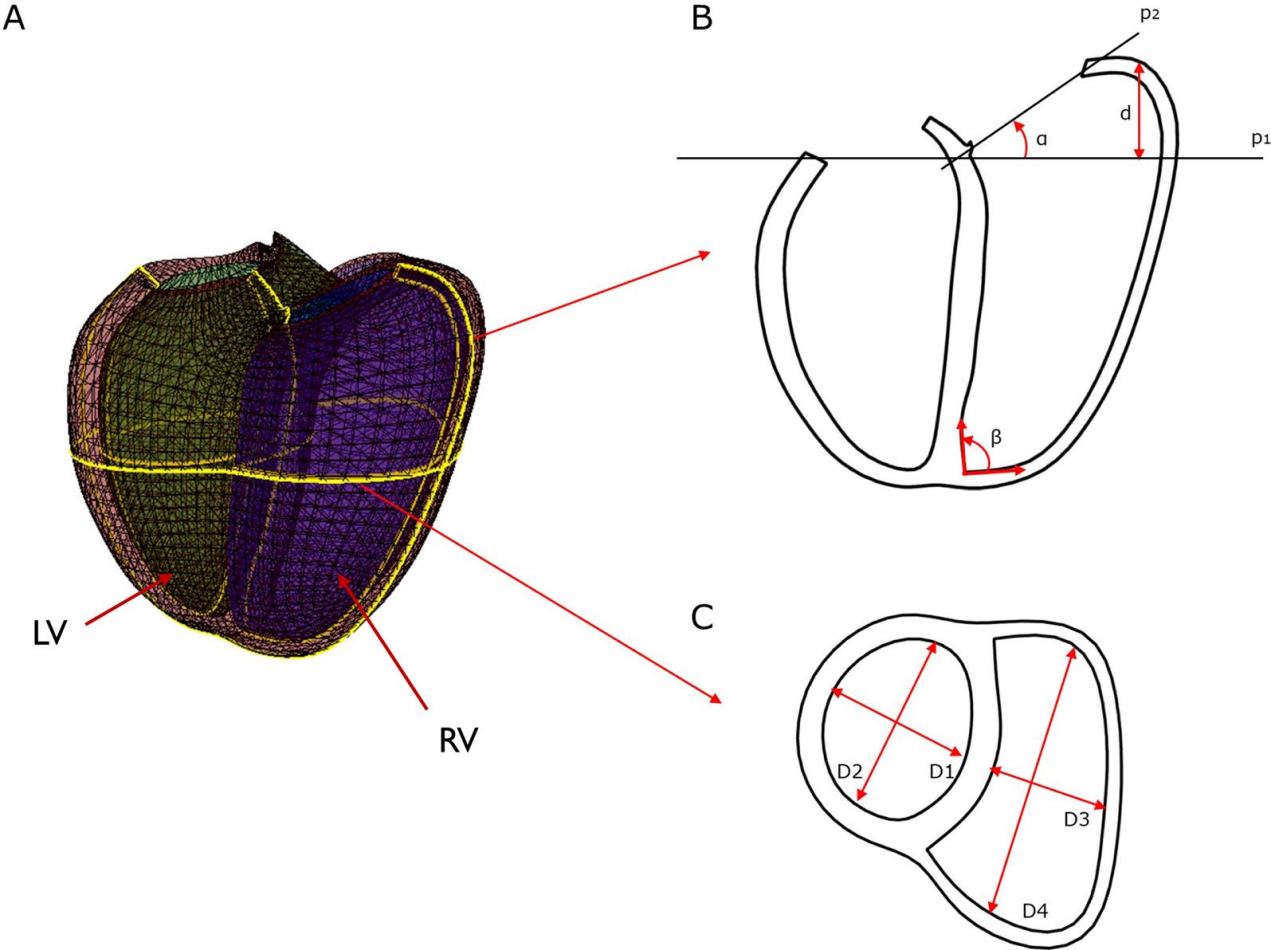
Model-based LV and RV dimension calculations. Green: LV; Purple: RV; Maroon: Epicardium. **A** Biventricular model and intersecting planes. **B** Long axis plane showing how apical angle (β), tricuspid tilt (α), and basal bulge (d) were calculated. **C** Mid-ventricular short axis view showing LV and RV lateral to septal dimensions (D1 and D3 respectively) and RV and LV anterior–posterior dimensions (D2 and D4 respectively)

Regional shape variations associated with PRVI were confirmed using univariate regression models for specific features derived from the 3D patient-specific biventricular models. These included: basal bulge, tricuspid tilting, apical dilation, RV and LV anterior–posterior dimensions and lateral to septal dimensions (Fig. 2). Basal bulge was calculated as the distance between the most basal point on the RV free-wall and its perpendicular projection onto a plane perpendicular to the LV long axis and going through the mitral valve centroid. Tricuspid tilt was calculated as the angle between a plane perpendicular to the LV long axis and going through the centroid of the mitral valve and a plane fitted to the tricuspid valve points, similar to [36]. Apical dilation was quantified using the apical angle. Apical angle was defined as in [37], using two lines defined at the RV apex in a four-chamber view: one aligned with the endocardium of the septum and another line aligned parallel to the most linear portion of the RV free wall endocardium. LV and RV lateral to septal dimensions were defined as the length of the minor axis perpendicular to the interventricular septum at mid-ventricle in both LV and RV. LV and RV anterior–posterior dimensions were defined as the length of the minor axis parallel to the interventricular septum at mid-ventricle in both LV and RV. Figure 2 summarizes how those remodeling features were calculated.

### Geometric strain

To further study interventricular relationships, model-derived systolic deformations for both chambers were assessed. Geometric strain was defined as the change in geometric arc length from ED to ES. Longitudinal strain (LS), which represents the longitudinal shortening of the cardiac muscle, and circumferential strain (CS), shortening along the circular perimeter, and radial strain (RS), thickening of the wall during systole, were derived from length changes between ED and ES using the Cauchy strain formula:

$$\varepsilon = \frac{L- {L}_{o}}{{L}_{o}}$$

where ε is the engineering strain, L the length at ES and L_0_ the length at ED. Changes in geometric arc length have previously shown good agreement with myocardial strain derived from tagging and displacement encoded CMR[38, 39]. For RS, myocardial wall thickness was calculated using a modified version of the centerline method [40] using the intersection between short axis slices and the biventricular model. For CS and LS, arc lengths were calculated from the model mesh. RV free wall and LV RS and CS were averaged over three different levels: base, mid-ventricle and apex. LV and RV LS were averaged over the apical 4-chamber and RV outflow tract views. The interventricular septum was included in the LV strain.

Univariate regression models were used to quantify the association between LV and RV strains and the amount of regurgitation. Although systolic CS and LS are conventionally negative, their absolute values were used in this study for simpler interpretation of the association with PRVI.

### Statistical analysis

Statistical analysis was carried out with R [41] (R Foundation for Statistical Computing, Vienna, Austria). All data are reported as mean and standard deviation or median and interquartile range, depending on the distribution, for continuous variable and as frequency for categorical variables. Normality was tested using Shapiro–Wilk tests. All variables were standardized before regression. Statistical differences were presented by p-values using one-way ANOVA or Kruskal–Wallis test depending on the distribution. A p-value of 0.05 was considered significant for the overall effect. Parameter estimates (PE), which represent the change in the response variable associated with a 1-unit change of the predictor, were used to measure the strength of the association between strain measurements, morphometric scores, and pulmonary regurgitation.

## Results

### RVLV model customization

All models were successfully customized to the manual contours and landmarks. Table 2 shows the mean volume and mass of LV and RV calculated from the biventricular models by numerical integration; they had good agreement with those calculated from the manual contours by short axis slice summation. Inter-observer errors in manual contour results in 35 participants are also reported in Table 2. The differences between model and manual estimates were similar to the inter-observer differences. The larger model RV volumes may be due to inaccuracies of short axis slice summation at the base and apex (in particular due to the basal bulge common in rTOF) since the biventricular model incorporates information from the long axis contours as well as the short axis contours. The larger LV mass in the model may be due to differences in the definition of LV vs RV myocardial partitions in the model relative to the contours.

**Table 2:** Error between biventricular model estimates and slice summation of manual contours

	Differences	PPMCC	Values from 3D models	Values from manual contours	Interobserver error (n = 35)
RV EDVI (ml/m^2^)	9 ± 15	0.89	147 ± 14	137 ± 15	− 7 ± 12
RV ESVI (ml/m^2^)	6 ± 10	0.90	90 ± 27	84 ± 33	− 11 ± 15
RVEF (%)	0 ± 7	0.70	39 ± 7	39 ± 9	5 ± 5
RVMI (g/m^2^)	2 ± 6	0.74	42 ± 11	39 ± 9	− 2 ± 5
LV EDVI (ml/m^2^)	0 ± 7	0.90	78 ± 14	79 ± 16	− 1 ± 5
LV ESVI (ml/m^2^)	3 ± 6	0.88	42 ± 24	39 ± 20	− 1 ± 3
LVEF (%)	4 ± 8	0.74	50 ± 6	46 ± 9	1 ± 3
LVMI (g/m^2^)	10 ± 6	0.75	76 ± 14	66 ± 11	− 4 ± 3

Data are presented as mean difference ± std. dev. of the differences. *RV* right ventricle, *LV* left ventricle, *EF* ejection fraction, *EDVI* end-diastolic volume index, *ESVI* end-systolic volume index, *LVMI* left ventricular mass index, *PPMCC* Pearson product moment correlation coefficient, *RVMI* right ventricular mass index

### Principal components

**Fig. 3:**
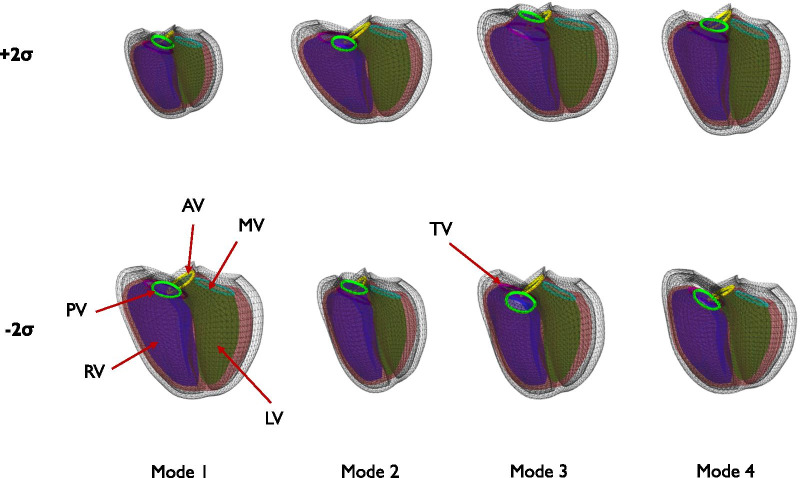
Principal component shape modes. Wireframe shows model at ED and shaded surfaces show the model at ES. Green: LV; Purple: RV; Maroon: Epicardium; Yellow: Aorta; Cyan: Mitral; Light green: Pulmonary; Pink: Tricuspid. Top rows show mean plus two standard deviations. Bottom row shows mean minus two standard deviations. *PV* pulmonary valve, *AV* aortic valve, *TV* tricuspid valve, *MV* mitral valve. See Additional file 1 for animations of these modes. To better visualize the change in function, motion was linearly interpolated between ED and ES shapes

The first four principal components of shape variation, accounting for the most variation in biventricular shape across the cohort (total 59%), are shown in Fig. 3 (anterior view). Animations of these shape variations can be found in Additional file 1. These represent the largest variations of biventricular shape as well as shape changes between ED to ES within the cohort. The first mode was associated with overall size and accounted for 37.5% of the total shape variance. The second mode accounted for 9.3% of the total shape variance and was associated with septal-free wall dimension (i.e., expansion and contraction of both ventricles toward and away from the interventricular septum) as well as the transition from a LV dominant apex shape to a RV dominant shape. The third mode (7.1%) was associated with basal vs apical bulging of the RV, with basal bulging associated with a tilt of the LV base away from the septum. The fourth mode (5.3%) was associated with a systolic septal deviation towards the RV, a common feature in rTOF remodeling due to volume overload [42, 43] in concert with LV diminution.

### Multivariate associations

The first 24 PCA modes accounted for 90.2% of the total shape variance and were selected to perform the multivariate regression. Morphometric scores were used as outcome (dependent) variables and sex, height, weight, age, degree of tricuspid regurgitation, and PRVI were used as predictor (independent) variables. The relationships between each predictor variable and the associated shapes were visualized using the method described in “Appendix”.

Height was a significant predictor in modes 1, 4, 9 and 15 (p < 0.001, p < 0.001, p = 0.005, and p = 0.04 respectively), accounting for 27% of the total variation. Weight was a significant predictor in modes 1 and 4 (p < 0.001 and p < 0.001 respectively, accounting for 16.3% of the total variation), age in modes 6, 9 and 14 (p = 0.01, p = 0.003, and p = 0.04 respectively) and sex in modes 1, 8, 9 and 14 (p = 0.003, p = 0.002, p = 0.006, and p = 0.04 respectively).

**Fig. 4:**
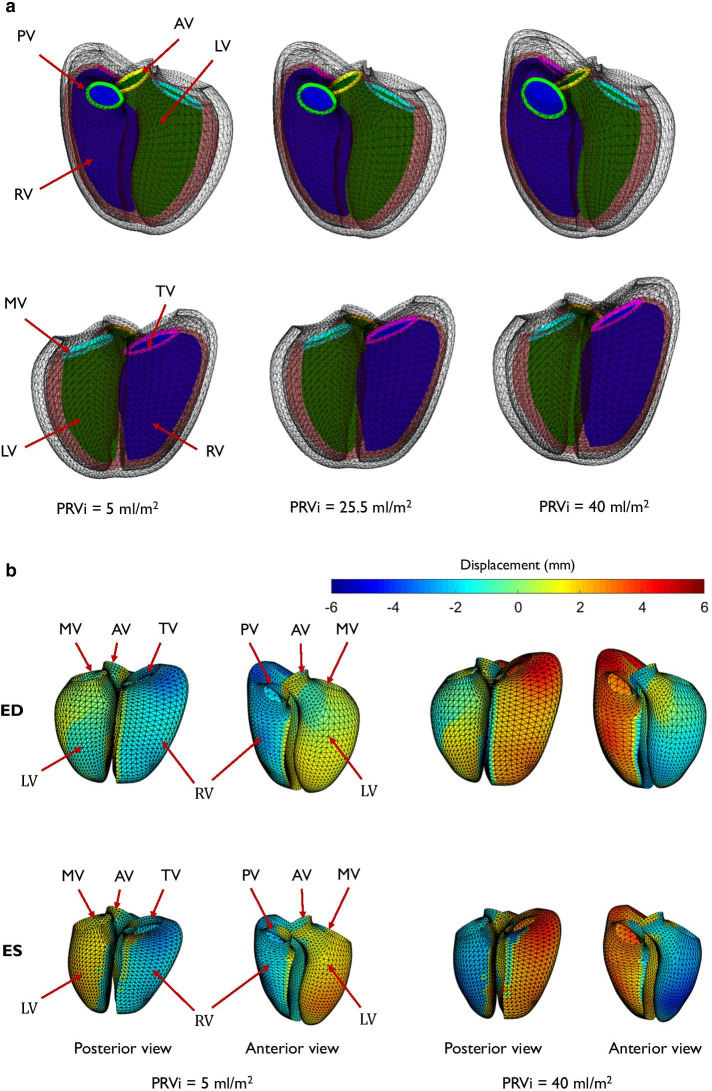
**a** Shape differences associated with increasing pulmonary regurgitant volume index. Green: LV; Purple: RV; Maroon: Epicardium; Yellow: Aorta; Cyan: Mitral; Light green: Pulmonary; Pink: Tricuspid. Top: anterior view; Bottom: posterior view. Left: mean shapes at 5 ml/m^2^; middle: mean shape at 25.5 ml/m^2^; right: mean shape at 40 ml/m^2^. PV: pulmonary valve, AV: aortic valve, MV: mitral valve and TV: tricuspid valve. For an animation of this variation see Additional file 2. To better visualize the change in function, motion was linearly interpolated between ED and ES shapes. **b** Endocardial surface colors show differences in shape from the mean for low (left) and high (right) PRVI. Scale bar is in mm. Red denotes displacement outward from the LV, and blue denotes displacement inward to the LV. Top: end-diastolic shape; bottom: end-systolic shape. *PV* pulmonary valve, *AV* aortic valve, *MV* mitral valve, *TV* tricuspid valve

Tricuspid regurgitation was significant predictor in modes 4 and 11 (p < 0.001 and p = 0.003 respectively). PRVI had significant effects on mode 1 (p < 0.001, PE = − 0.15), mode 2 (p = 0.007, PE = 0.3), mode 4 (p < 0.001, PE = − 0.4), mode 7 (p = 0.02, PE = − 0.27) and mode 8 (p = 0.002, PE = 0.3). Combining these scores, PRVI accounted for 12.3% of the total shape variation. A positive (negative) PE indicates increasing (decreasing) morphometric scores. For example, PRVI was associated with a negative coefficient for mode 1, meaning that as PRVI increased, patients had larger hearts. Figure 4a shows the regional shape differences associated with increasing PRVI computed with the regression model.

An animation of these shape differences can be found in Additional file 2. Figure 4b shows the quantitative differences in shape of the endocardial surfaces (expressed as a displacement in mm) relative to the mean PRVI of 25.5 ml/m^2^, at both ED and ES. Displacements outward from the LV are shown in red, displacements inwards towards the LV are shown in blue. The dilation of the RV outflow tract and apex are confirmed, as well as diminution of the LV. The associations between shape and PRVI were similar if height and weight were replaced by BSA in the predictor variables, and also if height and weight and age were replaced by BSA. The results were therefore robust to different methods of accounting for body habitus.

### Shape features

Results of the univariate regression models performed with specific shape features are summarized in Table 3. Significant correlations with PRVI are shown with corresponding PE (p < 0.05). In the RV, as PRVI increased, RV size increased, the RV apex dilated, and the tricuspid annulus tilted. In the LV, the distance between the LV free wall and LV septum reduced, resulting in a flattening of the LV. In addition, larger PRVI was associated with a systolic septal motion towards the RV (paradoxical septal motion), which is consistent with RV overload and decreased LV dimension at ED.

**Table 3:** Remodeling features derived from biventricular models

	Mean value	PRVI PE
Basal bulge at ED (mm)	16.5 ± 4.5	0.25
Basal bulge at ES (mm)	11.9 ± 3.4	
Tricuspid tilt at ED (°)	41.6 ± 12.39	
Tricuspid tilt at ES (°)	31.6 ± 10.9	0.25
Apical angle at ED (°)	85.7 ± 6.8	0.29
Apical angle at ES (°)	83.8 ± 7.5	0.21
LV lateral to septal dimension at ED (mm)	41.1 ± 6.4	− 0.25
LV lateral to septal dimension at ES (mm)	31.0 ± 6.0	− 0.27
RV lateral to septal dimension at ED (mm)	41.0 ± 6.7	0.24
RV lateral to septal dimension at ES (mm)	34.0 ± 6.0	
LV anterior–posterior dimension at ED (mm)	54.0 ± 7.7	
LV anterior–posterior dimension at ES (mm)	42.1 ± 6.9	− 0.18
RV anterior–posterior dimension at ED (mm)	71.1 ± 9.6	0.32
RV anterior–posterior dimension at ES (mm)	59.8 ± 8.7	0.26

*LV* left ventricle, *RV* right ventricle. Values are shown as mean ± SD. PRVI PE: pulmonary regurgitant volume index parameter effect for significant univariate correlations with parameters (p < 0.05). As normalized values were used in the regression, PRVI PE represents changes in standard deviations

**Fig. 5:**
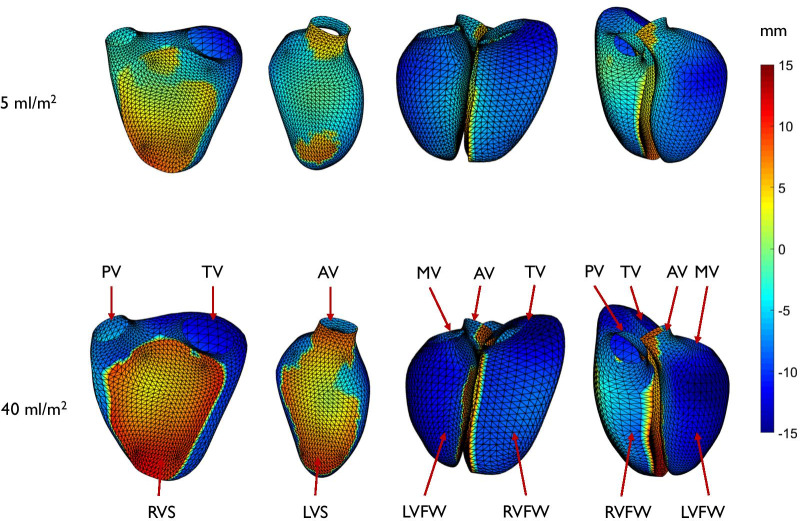
Changes in contraction patterns associated with increasing pulmonary regurgitation. Paradoxical septal motion is observed as regurgitation increases, as shown by an outward displacement of the septum toward the RV. *AV* aortic valve, *PV* pulmonary valve, *TV* tricuspid valve, *MV* mitral valve, *RVFW* right ventricular free wall, *LVFW* left ventricular free wall, *RVS* right ventricular septum, *LVS* left ventricular septum

To illustrate and quantify the paradoxical septal motion, Fig. 5 shows changes in systolic motion of the endocardial surfaces, expressed as a displacement in mm from ED to ES, for a PRVI of 5 ml/m^2^, and a PRVI of 40 ml/m^2^. Displacements outward from the LV are shown in red, displacements inwards towards the LV are shown in blue.

### Geometric strain

LS, CS, and RS for LV and RV are shown in Table 4 and significant correlations with PRVI are shown with corresponding PE. Although all RV strains were correlated with PRVI, for the LV only RS was significantly correlated with PRVI.

**Table 4:** Strain values derived from biventricular models

	LV LS (%)	LV CS (%)	LV RS (%)	RV LS (%)	RV CS (%)	RV RS (%)
Value	19.0 ± 5.7	20.6 ± 5.3	21.5 ± 7.4	20.7 ± 5.4	14.0 ± 3.8	20.8 ± 12.8
PRVI PE			0.32	0.22	0.25	0.37

*LV* left ventricle, *RV* right ventricle, *LS* longitudinal strain, *CS* circumferential strain, *RS* radial strain. Absolute values are shown for CS and LS for simpler interpretation of the association with PRVI

Values are shown as mean ± SD. PRVI PE: pulmonary regurgitant volume index parameter effect for significant correlations with strains (p < 0.05). As normalized values were used in the regression, PRVI PE represents changes in standard deviations

## Discussion

Adults with rTOF are exposed to ongoing physiologic sequelae of surgical interventions, causing remodeling which affects both the LV and RV. This cross-sectional study used atlas-based shape analysis to enable precise quantification of complex shape differences associated with pulmonary regurgitation. We used linear regression methods to control for confounding variables, and quantified independent effects associated with PRVI. PRVI explained more shape variance than any principal component except for the first (size mode), highlighting its importance in biventricular remodeling. Increasing PRVI was associated with dilation of the RV apex and outflow tract, together with a diminution of the LV and flattening in which the lateral free wall to septum distance decreased. Increasing PRVI was also associated with increased RV RS, LS and CS, as well as an increase in LV RS.

In the INDICATOR cohort study [44, 45], preoperative risk factors for postoperative occurrence of death or sustained tachycardia included RV systolic dysfunction, age at pulmonary valve replacement > 28, and elevated RV mass-to-volume ratio. Current guidelines recommend pulmonary valve replacement in asymptomatic patients if progressive RV dilation to RV end-systolic volume index (ESVI) > 80 ml/m^2^ and/or RV end-diastolic volume index (EDVI) > 160 ml/m^2^ is observed [46, 47]. However, optimal timing of replacement remains a challenge particularly in asymptomatic patients and there is wide qualitative institutional bias in practice. Owing to the continuity of muscle fibers between the LV and RV, a confined pericardial space and a relatively non distensible pericardium, volumes and function of one ventricle directly affect the other ventricle. Increased LVESVI and decreased LVEF have been associated with adverse outcomes in the INDICATOR cohort [45, 48] and other studies [49, 50]. Smaller LV diameters were previously associated with RV dilation and the severity of pulmonary regurgitation [15], as in the current study. PR has been associated with decreasing contribution of longitudinal shortening to the RV ejection and increasing lateral pumping, which results in larger volume changes and septal motion towards the RV [51], as seen in our data (Additional file 2). RV shortening has also been associated with exercise capacity in adult rTOF [52]. Fernandes et al*.* [16] also suggested that RV apical dilation alters LV geometry, thereby decreasing LV stroke volume. Alterations in LV geometry may also affect the mitral valve, contributing to the relatively high proportion of rTOF patients with mitral valve prolapse [17]. Our methods enable precise quantification of these relationships, including new features of LV flattening and motion, which highlight the importance of understanding the contributions of both RV and LV shape and their interactions.

The atlas captured regional shape differences and also reproduced global mass and volume with acceptable bias and precision (Table 2). Similar errors were previously obtained for 4329 cases from UK Biobank [28]. There was consistent overestimation of LV and RV mass in the model relative to manual contour estimations, likely due to differences in the apex and base. The biventricular model numerically integrated the volume contained within the 3D surfaces using information from both long and short axis images, whereas contour slice summation was performed only on short axis images, in which the contours are often difficult to estimate at the apex and base.

Shape models and atlas-based methods have previously shown RV shape changes associated with PR [12, 17, 36, 53]. These studies demonstrated RV dilation, basal bulging, tricuspid tilting, and apical dilation associated with pulmonary regurgitation, in agreement with the current study (Additional file 2, posterior view). This tilting may be a specific effect of RV dilation that contributes to tricuspid regurgitation, which develops in one third of rTOF patients [54]. Our biventricular analysis enabled extension of these methods to quantify the associations between biventricular shape and function and amount of PR.

In the German Competence Network rTOF study, reduced LV CS and RV LS were independent predictors of adverse outcomes [55]. Decreased LV LS has also been associated with adverse events [56]. In our study, we found that LV RS as well as RV RS, CS and LS increased as pulmonary regurgitation increased. A compensatory increase in RV free wall RS in response to PR and volume overload was also observed in [51, 57], which may be due to increased RV SV and a higher preload. The increase in LV RS found the current study may be due to the inclusion of the septum into the LV RS measurement.

Our study also found a relationship between RV longitudinal shortening and the amount of regurgitation, in agreement with Ylitalo et al. [18]. However, conclusions regarding the effects of PR on longitudinal shortening have varied between studies. In pediatric populations, Ylitalo et al. [18] found the PR led to an increase in RV LS, on the other hand, Eyskens et al*.* [58] and Ouyang et al*.* [59] found an inverse correlation between PR and RV LS measurements. Studies in adult rTOF patients did not show any variation in RV and LV strain in relation to PR [16, 22, 43]. However, reduced contractile function in relation to PR was demonstrated in [3] but did not have any influence on RV strain measurements. Our study contained mostly younger adult patients, but decreasing strain beyond a certain point may occur with more severe disease.

The type of primary repair has also been shown to affect RV shape [60]. Zaidi et al*.* [60] found differences in volume and regional curvatures in patients with transannular repair vs those with pulmonary valve preserving repair. In our study, 78.4% had transannular patch primary repair. Also, our findings of reduced septal-lateral dimension are consisted with increased curvature in the free wall.

Although data sharing mechanisms provided through the CAP enable merging of data from different institutions, access to clinical information was limited. Data on ethnicity was not available. Indices of RV pressure loading were not available, and investigation of pressure effects requires further study, ideally using measurements from catheter recordings. The interpretation of strain measurements in the rTOF population is also difficult due to the competing effects of increased force of contraction with higher preload, and decompensating RV contractility. Another limitation of the study is the use of cross-sectional data. The temporal evolution of ventricular dysfunction in relation to regurgitation should be performed in longitudinal studies. Finally, our study did not quantify dyssynchrony (due to right bundle branch block) or late gadolinium enhancement patterns, which may be have important effects on biventricular function [61, 62].

Future application of these methods will examine quantitative shape changes in relation to interventions and clinically significant metrics, including pulmonary valve replacement and adverse outcomes. The distillation of complex shape features into a small number of morphometric scores allow precise quantification of mechanistic effects linking structural and functional alterations to a variety of clinical measures, including rhythm disturbances, functional score, restrictive RV physiology, and exercise capacity. Relationships between morphometric scores and future adverse outcomes will enable examination of mechanisms of developing risk. Fully automated analysis including quantification of contours, biventricular shape model customization, and computation of scores relative to reference populations, is now possible using machine learning and AI methods.

## Conclusions

Biventricular morphometric relationships with important clinical features can be quantified in rTOF patients. Increasing PR is associated with LV diminution and septal flattening, in conjunction with RV dilation, especially at the base and apex, as well as an increase in RV systolic strain. Specific and quantifiable biventricular shape characteristics, revealed in biventricular models of rTOF, can be used to quantitatively evaluate RV and LV dysfunction in these patients. This study provides a basis for including quantitative shape analysis into the clinician’s toolbox, providing simple scores which express complex features that experienced clinicians may well recognize but which have been difficult to report until now.

### Supplementary Information

**Additional file 1.** Animation of the first four PCA components. Left: anterior view. Right: posterior view. “s” indicates the number of standard variations.

**Additional file 2.** Shape changes due to pulmonary regurgitation. Left: anterior view. Right: posterior view. “s” indicates the number of standard variations.

## Data Availability

Data and shape models are available from www.cardiacatlas.org.

## Appendix

Each shape model was subdivided into 5,810 points on the LV and RV endocardial and epicardial surfaces as well as the four valves. These points (x,y,z) coordinates at ED and ES were concatenated into a single shape vector with 34,860 elements. The shape vectors from each patient were assembled into a data matrix (one row per case). The PCA shape modes were calculated from the covariance matrix and morphometric scores for each patient were calculated by projection onto the shape modes and then standardized to provide z-scores. A multivariate linear regression was performed to estimate the contribution of predictor variables to each principal component score:

$${\text{T}}\, = \,{\text{AB}}\, + \,{\text{R}}.$$

where T are the morphometric scores, A is a matrix of predictor variables, B is a matrix of regression coefficients and R is a residual matrix. The predictor variables comprised sex, age, height, weight, and PRVI. For each predictor variable, the associated shape changes were visualized by calculating the contribution to the morphometric scores for that predictor.

## Abbreviations

BSA: Body surface area
CAP: Cardiac atlas project
CMR: Cardiovascular magnetic resonance
CS: Circumferential strain
ECG: Electrocardiogram
ED: End-diastole
EDVI: End-diastolic volume index
EF: Ejection fraction
ES: End-systole
ESVI: End-systolic volume index
LS: Longitudinal strain
LV: Left ventricle/left ventricular
LVEF: Left ventricular ejection fraction
LVM: Left ventricular mass
LVMI: Left ventricular mass index
PA: Pulmonary artery
PC: Phase contrast
PCA: Principal component analysis
PE: Parameter estimates
PR: Pulmonary regurgitation
PRVI: Pulmonary regurgitant volume index
RS: Radial strain
rTOF: Repaired tetralogy of Fallot
RV: Right ventricle/right ventricular
RVM: Right ventricular mass
RVMI: Right ventricular mass index
SV: Stroke volume
