# Supplementary material for: Right-left ventricular shape variations in tetralogy of Fallot: associations with pulmonary regurgitation
Source: J Cardiovasc Magn Reson. 2021 Oct 7;23:105. doi: 10.1186/s12968-021-00780-x (PMC8496085; doi:10.1186/s12968-021-00780-x)
Supplement: Supplementary file 1 — Additional file 1. Animation of the first four PCA components. Left: anterior view. Right: posterior view. “s” indicates the number of standard variations. [file 12968_2021_780_MOESM1_ESM.pptx]

## Slide 1
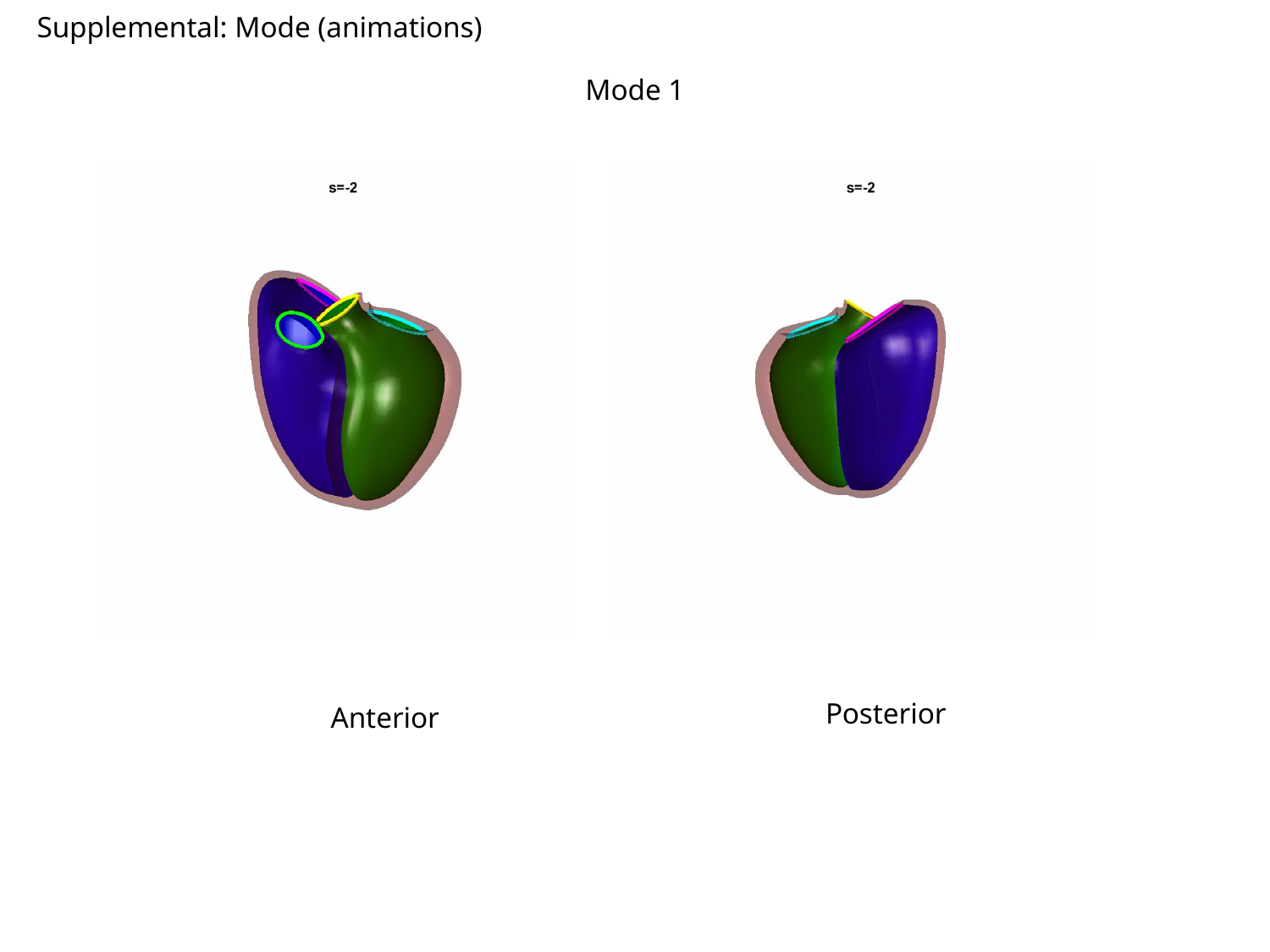

Supplemental: Mode (animations)
Mode 1
Posterior
Anterior

## Slide 2
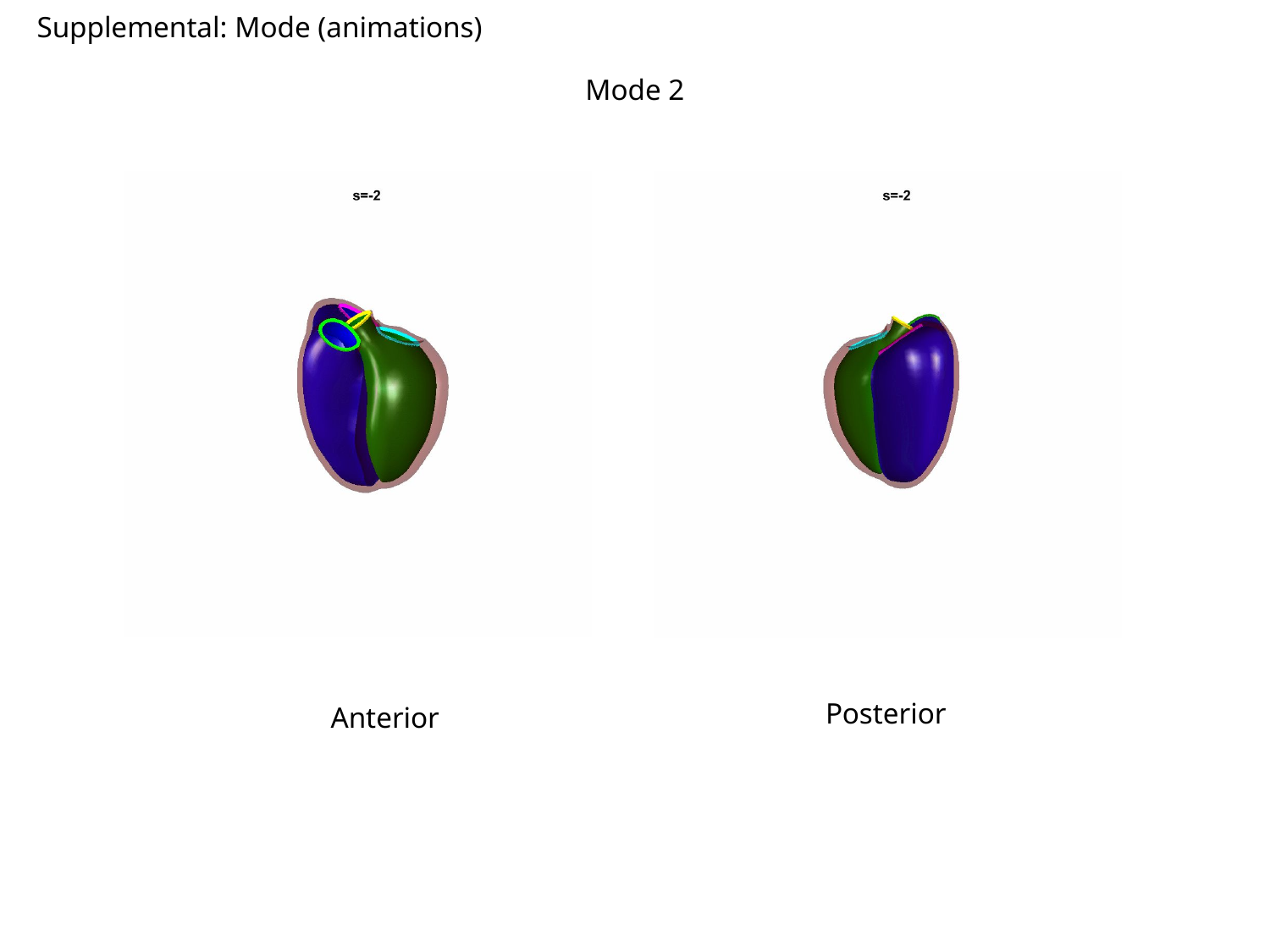

Supplemental: Mode (animations)
Mode 2
Posterior
Anterior

## Slide 3
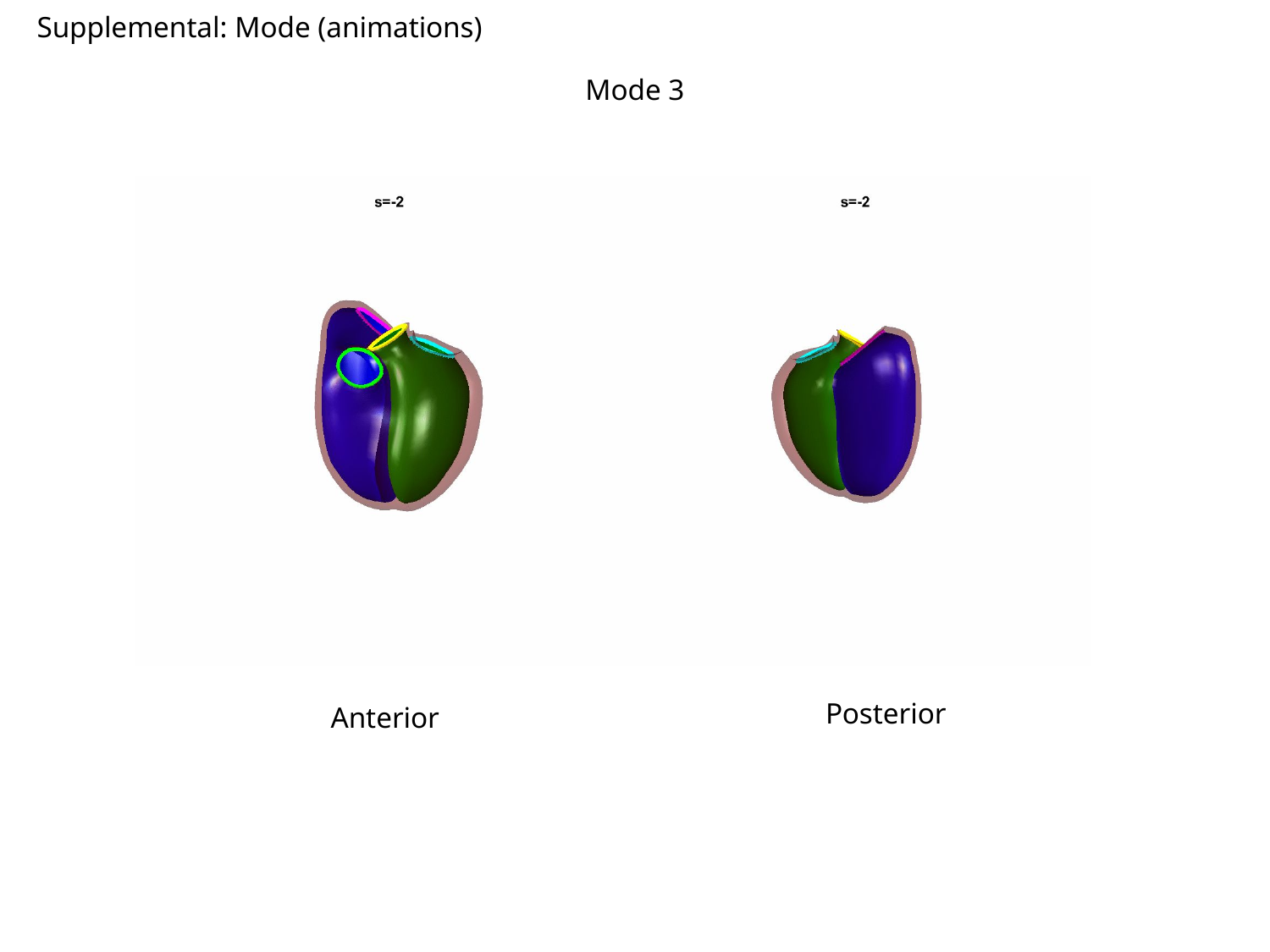

Supplemental: Mode (animations)
Mode 3
Posterior
Anterior

## Slide 4
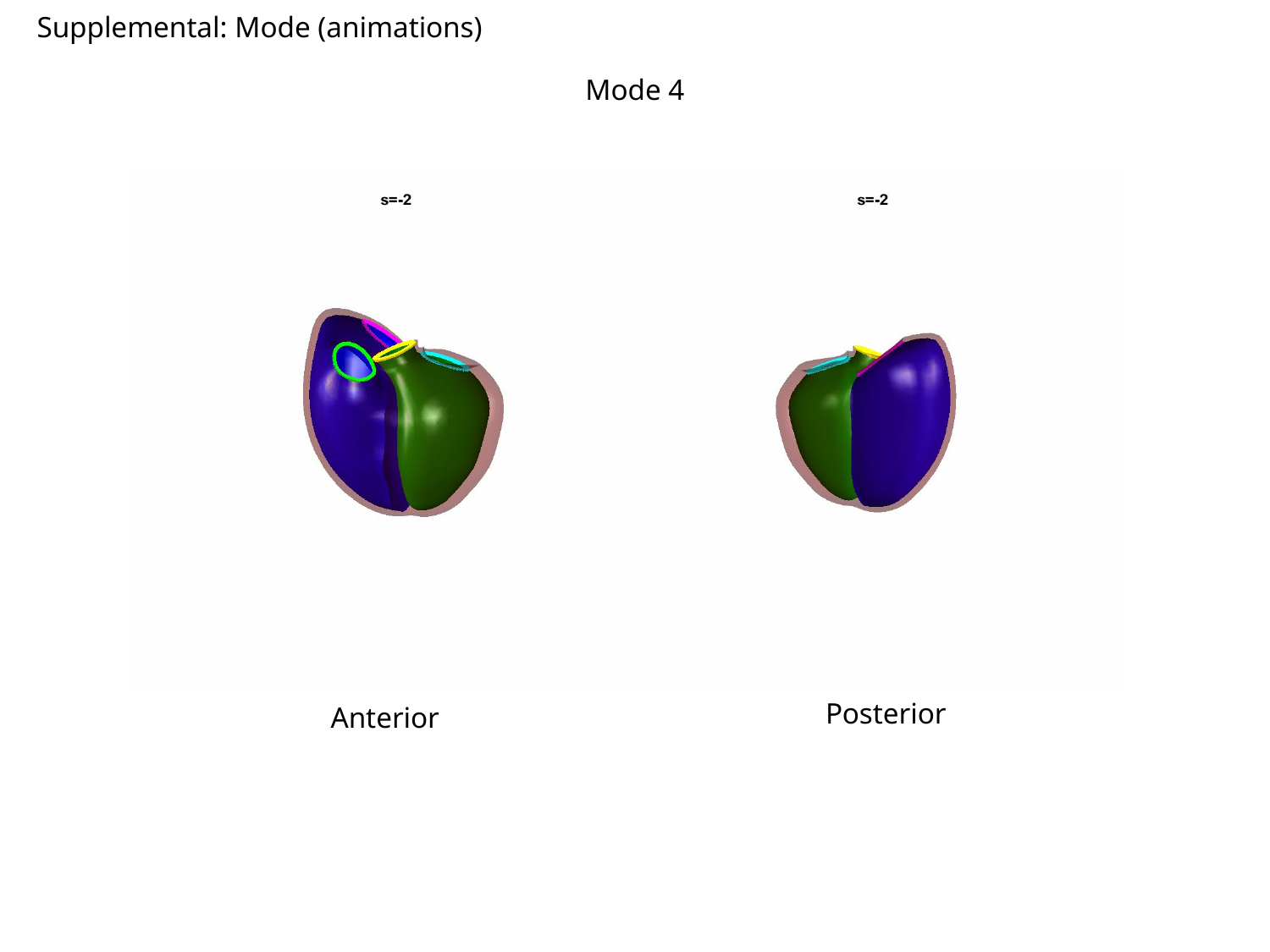

Supplemental: Mode (animations)
Mode 4
Posterior
Anterior
