# Supplementary figures and images for: Right-left ventricular shape variations in tetralogy of Fallot: associations with pulmonary regurgitation
Source: J Cardiovasc Magn Reson. 2021 Oct 7;23:105. doi: 10.1186/s12968-021-00780-x (PMC8496085; doi:10.1186/s12968-021-00780-x)

## Slide 1
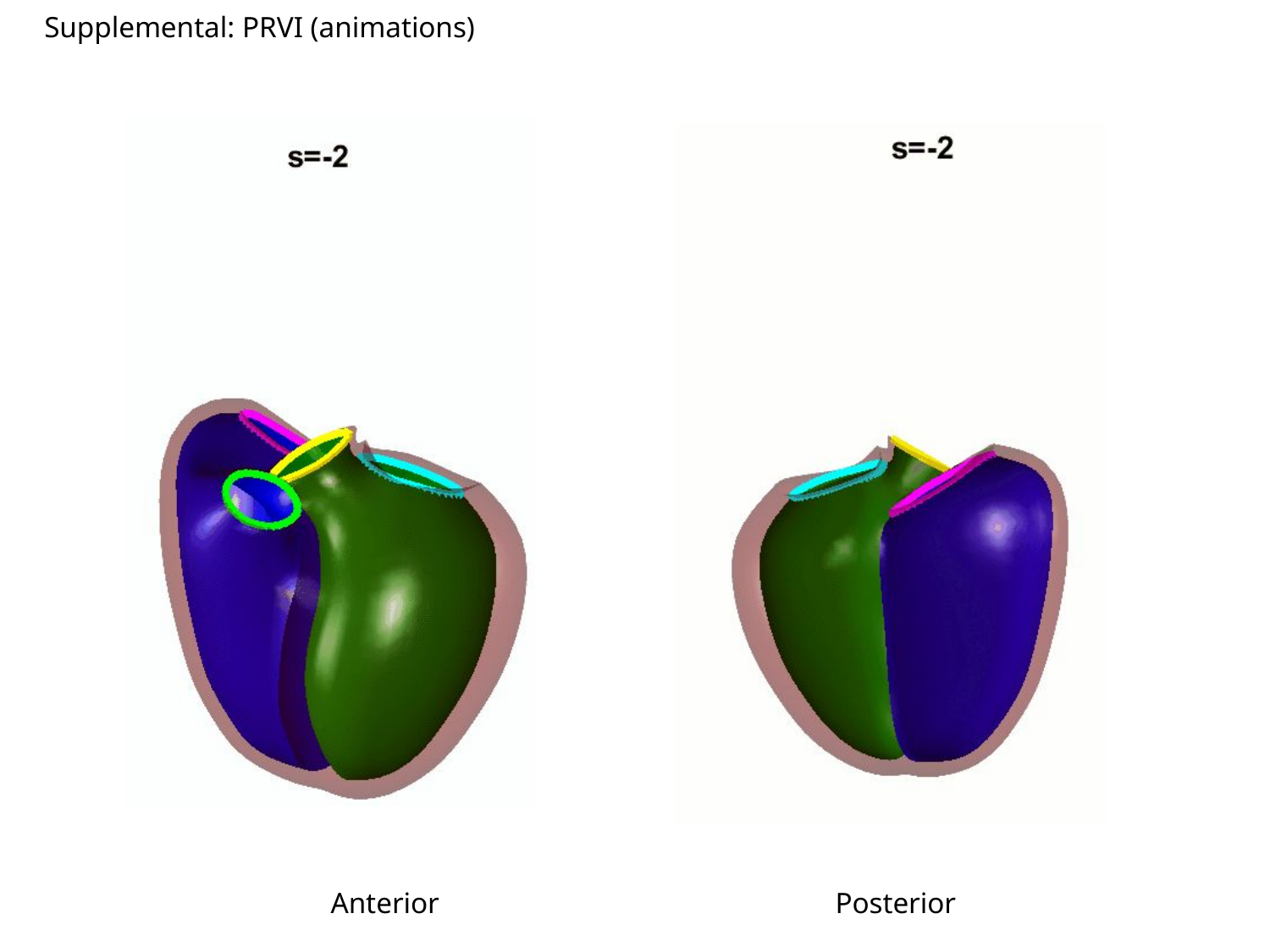

Supplemental: PRVI (animations)
Anterior
Posterior

Supplement: Supplementary file 2 — Additional file 2. Shape changes due to pulmonary regurgitation. Left: anterior view. Right: posterior view. “s” indicates the number of standard variations. [file 12968_2021_780_MOESM2_ESM.pptx]
